# High and equitable mass vitamin A supplementation coverage in Sierra Leone: a post-event coverage survey

**DOI:** 10.9745/GHSP-D-12-00005

**Published:** 2013-06-26

**Authors:** Mary H Hodges, Fatmata F Sesay, Habib I Kamara, Mohamed Turay, Aminata S Koroma, Jessica L Blankenship, Heather I Katcher

**Affiliations:** a Helen Keller International, Freetown, Sierra Leone; b Ministry of Health and Sanitation, Freetown, Sierra Leone; c Helen Keller International, Regional Office for Africa, Nairobi, Kenya

## Abstract

**Background:**

In Sierra Leone, children ages 6–59 months receive twice-yearly vitamin A supplementation (VAS) through Maternal and Child Health Week (MCHW) events. VAS coverage in 2011 was calculated using government tally sheets of vitamin A capsule distribution and outdated population projections from the 2004 census. We conducted a national post-event coverage (PEC) survey to validate coverage and inform strategies to reach universal coverage of VAS in Sierra Leone.

**Methodology:**

Immediately following the November 2011 MCHW event, we conducted a national PEC survey by interviewing caregivers with children ages 6–59 months using a randomized 30X30 cluster design (N = 900). We also interviewed one health worker and one community health worker in each cluster to determine their knowledge about VAS (N = 60).

**Results:**

VAS coverage was 91.8% among children ages 6–59 months, which was lower than the 105.1% reported through tally sheets. Coverage was high and equitable among all districts and between age groups (98.5% for infants ages 6–11 months and 90.5% for children ages 12–59 months). Major reasons for not receiving VAS were that the child was out of the area (42.4%), the household was not visited by community health workers (28.0%), and the caretaker was not aware of the event (11.9%).

**Conclusion:**

Twice-yearly delivery of VAS through MCHW events achieved consistently high and equitable coverage in Sierra Leone. Universal coverage may be achieved through continued focus on communication and targeted outreach to hard-to-reach areas during the MCHWs.

## Abstract (precis)

In Sierra Leone, an intensive mass vitamin A supplementation (VAS) campaign to reduce under-5 mortality reached over 90% of children ages 6–59 months, eliminating coverage disparities among districts and between age groups. Delivering VAS with other essential maternal and child health interventions was key to the success.

## BACKGROUND

Vitamin A supplementation (VAS) is one of the most cost-effective child survival strategies in areas where vitamin A deficiency (VAD) exists.^1^-^2^ Strong evidence shows that in settings where VAD is prevalent, twice-yearly receipt of VAS by at least 80% of children ages 6–59 months reduces risk of mortality from measles by an average of 50%, from diarrhea by an average of 40%, and from all causes by 24%.^3^-^4^ A study in India carried out in a programmatic setting did not demonstrate such impact, but the study and related expert comments underscore the important of validating VAS coverage data to drive programs.^5^-^6^

VAS delivery through integrated events, such as Maternal and Child Health Weeks (MCHWs), is the most efficient method for reaching high and equitable coverage. Over the past 15 years, roughly 80 countries have scaled up vitamin A interventions, including 41 of 45 priority countries in sub-Saharan Africa that have high under-5 mortality rates and/or high VAD prevalence.^7^

Integrating VAS campaigns with other essential maternal and child health interventions provides maximum efficiency and coverage.

In 1999, Sierra Leone had one of the highest under-5 mortality rates in the world (242 per 1,000 live births)^8^ and an estimated VAD prevalence of 47% among children ages 6–59 months.^9^ That year, the Ministry of Health and Sanitation (MoHS) initiated large-scale VAS as part of its strategy to reduce child mortality.^10^-^11^ Although locally produced palm oil (rich in carotenes, which are vitamin A precursors) is widely available in Sierra Leone, its use in infant and young children's diets appears limited.

The Ministry estimated VAS coverage from events implemented between 1999 and 2003, but they were based on tally sheets using outdated 1985 census data. To better assess VAS coverage and inform strategies to improve coverage nationally and among hard-to-reach populations, the MoHS, Helen Keller International (HKI), and the United Nations Children's Fund (UNICEF) implemented a **post-event coverage (PEC) survey in 2004.** According to the survey, VAS coverage among children 6–59 months of age was 68.2% compared with the reported 83.0% coverage based on tally sheets, with large disparities found among districts.^12^ Coverage also varied between age groups, with only 41% of infants ages 6–11 months receiving VAS compared with 73% of children ages 12–59 months.

As a result of these findings, the MoHS, with technical support from HKI and UNICEF, revised their distribution plan for VAS events at both national and district levels and launched an intensive communication campaign targeting caregivers and health care workers. In addition, the MoHS Nutrition Department and the Expanded Programme on Immunizations (EPI) strengthened their partnership, so that VAS, catch-up vaccinations, and deworming were integrated into twice-yearly MCHWs. The aim of the MCHWs was to deliver a package of child survival interventions with universal coverage among children 6–59 months of age.

To measure the impact of the refined strategy, the partners implemented a **follow-up PEC survey in 2005.** National VAS coverage among children 6–59 months of age had increased from 68.2% in 2004 to 95.0% in 2005, with high coverage across districts.^12^ Additionally, coverage disparities between age groups had declined, with 91.8% of infants ages 6–11 months and 95.5% of children ages 12–59 months receiving VAS.

Since 2005, high VAS coverage has been sustained, with over 80% coverage consistently obtained in all districts as measured by tally sheets (based on 2004 census projections). The sustained high coverage of VAS and other essential child survival interventions may have contributed to under-5 mortality reduction in the country, from 214/1,000 live births in 2005 to 200/1,000 in 2008 and to 185/1,000 in 2011.^8^

These major reductions in child mortality indicate that the measure of VAS coverage by tally sheets—while based on census projections from 2004—is still imprecise as it uses the 2005 child mortality rate of 214/1,000 to determine the projected population.

To validate VAS coverage and inform strategic planning of the MCHWs, the MoHS and HKI conducted a **national PEC survey immediately after the November 2011 MCHW.** The survey used EpiSurveyor technology (currently called Magpi; https://www.magpi.com/) to collect data through mobile phones on VAS coverage and characterize the children who did not receive VAS during the November 2011 MCHW. The survey also evaluated the level of VAS awareness among caregivers and health care workers, including from where caregivers heard about VAS, to evaluate the quality of MCHW social mobilization activities. Coverage data from the PEC survey was compared with tally sheet data and population projections to determine the reliability of administrative data.

This paper discusses the 2011 PEC survey and how the findings will inform VAS program design in Sierra Leone.

## METHODOLOGY

### MCHWs and Mass Vitamin A Supplementation

Mass VAS for the November 2011 MCHW was conducted over 5 days using mixed delivery methods, including fixed distribution points by health workers (HWs) and door-to-door distribution by teams of community health workers (CHWs) consisting of one distributor and one record keeper. Other interventions administered concurrently included deworming with albendazole of children ages 12–59 months, screening and referral of underweight children using mid-upper arm circumference (MUAC), delivery of tetanus toxoid for women of childbearing age (15–49 years), and testing and management of pregnant women with HIV infection to prevent mother-to-child transmission (PMTCT). HWs and CHWs collected and reported daily tallies of vitamin A capsules distributed to primary health units (PHUs), District Health Management Teams (DHMTs), and the EPI Unit of the MoHS.

### PEC Sampling, Sample Size, and Survey Sites

We adapted the World Health Organization-EPI (WHO-EPI) cluster sampling methodology to determine the sample size for the PEC survey.^13^-^14^ Using probability proportional to size sampling (PPS) methodology, we randomly selected 30 enumeration areas (EAs) representing all 14 districts in Sierra Leone, with support from the National Statistics Office using population data from the 2004 national census.^13^

Of the 30 EAs sampled, 11 were in the north, 7 in the east, 6 in the south, and 6 in the west. In each EA, we selected 30 households by going to a central location (for example, a church, mosque, or communal center), spinning a pen, counting the number of houses to the end of the cluster in the direction the pen pointed, and selecting a random house to start. In addition, we randomly sampled and interviewed one HW from the nearest health facility and one CHW in each EA (N = 60).

Caregivers were eligible to participate in the survey if they had a child who was 6–59 months old in November 2011 (N = 900). If there was more than one caregiver in the household, we chose one at random by writing down each eligible caregiver's name on a slip of paper, putting the paper slips in a hat or basket, and selecting one at random. If the caregivers had multiple children ages 6–59 months, we randomly selected one child to be the focus of the interview using the same methodology that we used for randomly selecting caregivers. We verified children's ages with health cards whenever possible. When health cards were not available, we estimated children's ages using a local event calendar.

We selected HWs and CHWs based on their availability at the nearest PHU. When there was more than one HW or CHW present, we randomly selected one using the simple ballot system described above.

### Questionnaire Development and Training of Enumerators

We developed a standard, coded survey for caregivers and another for both HWs/CHWs and translated both into 3 local languages: Krio, Mende, and Themne. We programmed the English version of the questionnaire into Nokia X2-01 mobile phones using the EpiSurveyor mobile phone program.

Ten enumerators attended a one-day training session on vitamin A, VAS, PEC survey methodology, the survey questions, and EpiSurveyor. We paired the enumerators into 5 teams, with 2 teams conducting the PEC survey in the north and one team each conducting the PEC survey in the east, south, and west regions.

Each team of enumerators received 2 Nokia X2-01 mobile phones to enter data, hard copies of the questionnaires, chalk to mark the houses visited, and vitamin A capsules to help mothers recall VAS receipt. Surveyors marked each house with a code:

✓ PECS for a completed house


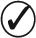
 PECS for a house to be revisited

N = PECS for a house with no eligible children

The enumerators first recorded the data onto hard copies of the questionnaires and then subsequently entered the data into the mobile phones at the end of each day. We deemed it safer in remote, hard-to-reach sites for enumerators to use hard copies rather than to use the mobile phones in public places. The EpiSurveyor account administrator cross-checked the data on the hard copies with the database data before analysis to ensure quality control. Enumerators completed data collection within one week, and we held a debriefing session a day later with them to get feedback on their challenges with conducting the PEC survey and with using EpiSurveyor.

### Ethical Considerations

We did not offer compensation or any other incentive to survey respondents. To protect the confidentiality of respondent information, the enumerators did not record names or addresses. The MoHS responsible for medical research and ethics approved the study monitoring. The enumerators made courtesy calls to traditional village chiefs to explain the purpose of the PEC survey and to obtain their permission to conduct the survey. Because literacy rates are low in Sierra Leone,^15^-^16^ we obtained informed verbal consent from caregivers, HWs, and CHWs and interviewed only those who gave their consent to participate in the study.

### Statistical Analysis

All data were downloaded from mobile phones to the EpiSurveyor program, exported to Microsoft Excel, and then cleaned by comparing the uploaded data to the responses on the hard copies. Frequencies and percentages were calculated to estimate VAS coverage and chi-squared tests were conducted to test for differences between groups. Proportions for caregiver data were weighted using the inverse of the probability of selecting a household within a cluster. Because the selection and clusters were done using PPS, sample weighting was implemented only at the household level. All data were analyzed using SPSS^®^ Version 20.

## RESULTS

### Demographic Characteristics of Survey Respondents

We interviewed a total of 900 caregivers, 26 HWs, and 34 CHWs. Of the 900 caregivers sampled, we could not confirm the exact age for 5 children (0.6%) from the enumerators' forms. In addition, we determined that 16 children (1.8%) were under 6 months of age at the time of the MCHW based on their birth date or estimation of age by the local calendar of events. Of the 879 children confirmed to be 6–59 months during the November MCHW, 16% were 6–11 months old and 84% were 12–59 months old (see Table).

**TABLE.** Demographic Characteristics of Sampled Children and Caregivers

Characteristics	Male	Female	Total
Age, n (%)			
6–11 months	81 (57.0%)	61 (43.0%)	142 (16.2%)
12–59 months	362 (49.1%)	375 (50.9%)	737 (83.8%)
Religion, n (%)			
Muslim	277 (49.3%)	285 (50.7%)	562 (63.9%)
Christian	164 (52.2%)	150 (47.8%)	314 (35.7%)
Prefer not to say	2 (66.7%)	1 (33.3%)	3 (0.3%)
Total, n (%)	443 (50.4%)	436 (49.6%)	879 (100%)

There was no significant difference between the number of boys (50.4%) and girls (49.6%) selected as the focus child of the interviews with caregivers. Of the 879 caregivers interviewed, 80% were mothers of the selected child, 11% were grandmothers, 4% were fathers, 1% were siblings, and 4% were other relatives. There were significantly more Muslim (64%) than Christian caregivers (36%) (*P* = 0.001). Caregivers had child health cards in 86% of households.

Of the CHWs and HWs interviewed, 23 (38%) were men and 37 (62%) were women. Nearly half had more than 5 years of health work experience, one-third had 3–5 years of experience, and about 18% had 1–2 years of experience.

### VAS Knowledge Among Caregivers and Health Care Workers

When asked about the benefits of VAS, caregivers said that it prevents sickness (42%), prevents blindness (22%), improves growth (9%), and reduces risk of death (6%). Of the caregivers surveyed, 24% correctly stated the first age at which infants should receive VAS (6 months), and 15% correctly stated how often VAS should be received (every 6 months until 59 months or at every MCHW). Major sources of information about VAS included HWs (38%), radio (30%), and CHWs (27%), with TV, posters, and friends and family listed as minor sources.

Caregivers received information about VAS mostly through health care workers and the radio.

HWs and CHWs responded that VAS prevents sickness and/or improves health (67%), prevents blindness (53%), reduces risk of death (28%), and helps with vision (18%). The majority of HWs/CHWs correctly cited the age of first VAS (93%) and the VAS dosage for children ages 6–11 months and 12–59 months (85% and 83%, respectively).

Of the HWs/CHWs interviewed, 85% could correctly state how often VAS should be received. The major sources of information about VAS among HWs and CHWs were in-service training (67%) and job aids (10%). Minor sources of information included radio messages, pre-service training, and policy documents.

### VAS Coverage According to the PEC Survey

Overall, 91.6% of caregivers reported that their child received VAS during the last MCHW. Few caregivers (2.5%) could not remember if their child had received VAS, and 5.9% reported their child did not receive VAS during the last campaign. Coverage for infants 6–11 months old was 98.5% and for children 12–59 months old, 90.5%. While the study did not have enough power to measure coverage in each district, coverage in all selected clusters was considered high, ranging from 86.7% to 97.8%.

Of the 5.9% of caregivers who reported their children did not receive VAS:

- 42.4% had been out of the area at the time of the MCHW
- 28.0% had homes that were not reached by the teams
- 11.9% were not aware of the MCHW
- 17.7% cited other reasons: the child was sick, the head of the family had refused, the family was busy, or VAS supplies had run out

Programmatic barriers to receiving VAS include lack of health worker outreach to certain locations and lack of caregiver awareness about the campaigns.

Sources of VAS included:

- 94.1% via door-to-door
- 4.4% via health centers
- 0.8% at nursery schools
- 0.7% from mobile teams

### VAS Coverage Reported by the Ministry of Health

Based upon tallies of capsules utilized (1,266,861) and a target population projection from the 2004 census (1,205,077), VAS coverage reported by the MoHS for the November 2011 MCHW was 105.1%.

## DISCUSSION

VAS coverage during the November 2011 MCHW event was 91.6% based on results of the PEC survey. This level was not drastically lower than the 105.1% reported through MoHS tally sheets, suggesting that the higher VAS coverage reported through the tally sheets may be due to underestimation of the target population from inaccurate census projections. The 3 PEC survey rounds (2004, 2005, and 2011) were useful to validate VAS coverage through the MCHWs and to refine the planning and strategic development process of VAS events to ensure high coverage.

Validating administrative data with post-campaign surveys helps to refine planning, supply chain management, and implementation of future VAS campaigns.

Using the number of vitamin A capsules distributed as reported from tally sheets and the estimated coverage from the PEC survey, we estimate the actual target population of children 6–59 months of age as 1,369,579. This is 14% higher than the current target population of 1,205,077 used by the MoHS.

The modified population estimate is an important adjustment to ensure that the appropriate number of vitamin A capsules, albendazole, and vaccines for measles and polio are ordered for upcoming MCHWs to prevent shortages during the events. This may help to increase coverage, since in the PEC survey, 35% and 17% of HWs/CHWs reported a shortage of 100,000 and 200,000 IU capsules, respectively. PEC surveys will be necessary to measure future changes in coverage since administrative data is prone to over-reporting.^15^

### Success Factors to High VAS Coverage

The high VAS and deworming coverage during the MCHWs in Sierra Leone is likely the result of many factors, including monthly macroplanning meetings at the national level, sufficient funding and distribution of resources to enable VAS distribution in hard-to-reach areas, social mobilization involving high-level dignitaries as well as community leaders, and daily debriefings to enable rapid responses to weak performing areas (see Box).

**BOX. High VAS Coverage in Sierra Leone: Success Factors**

- Regular planning and coordination meetings by a MCHW taskforce, chaired by the Ministry of Health and starting at least 6 months prior to the MCHW
- Availability and use of microplanning tools
- Inclusion of MCHW into national and district workplans
- Use of a pre-verification questionnaire that is sent to all District Medical Officers (DMOs) to assess their district's preparedness for the MCHW
- Interactive trainings for supervisors, vaccinators, and distributors prior to the MCHW
- Use of door-to-door and temporary fixed-post strategies to reach target children
- Provision of fuel and transportation costs to DHMTs and national supervisors, as well as provision of boat hire for districts with riverine populations
- Extensive social mobilization including promotion by the President of Sierra Leone, the First Lady, Minister of Health, jingles, banners, press releases, radio discussions in 4 languages, announcements by town announcers, and performances by music and drama groups
- Supervision at all levels and daily meetings at the DHMT level during implementation to identify gaps and solve problems
- Post-event reporting of results and sharing lessons learned at district and national Task Force levels

Similar to other countries, integration of VAS with other child survival strategies, including measles and polio immunizations, deworming, and MUAC screening, has been successful in increasing VAS coverage.^17^ These interventions have been consistent over the years in Sierra Leone, making it difficult to single out the intervention that has had the greatest impact on mortality. It is likely that improvements in all child survival services have contributed to the decrease in under-5 mortality observed in Sierra Leone (see Figure).

**Figure:**
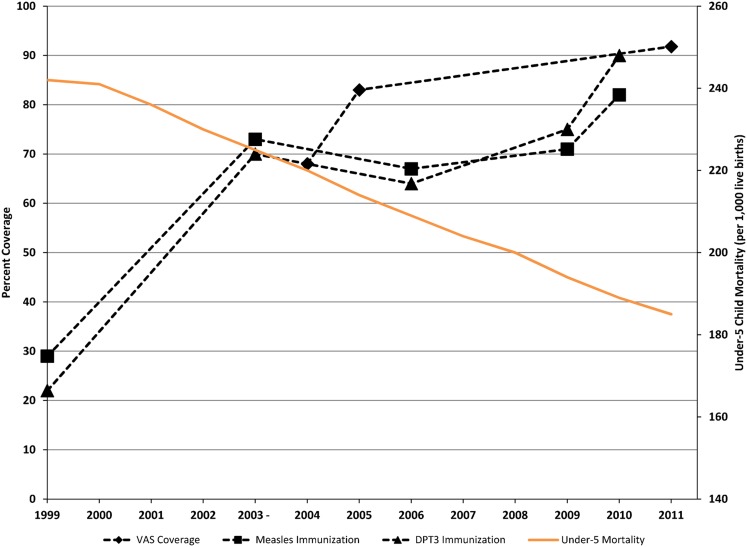
Changes in Under-5 Mortality and Coverage of Major Child Survival Interventions, Sierra Leone, 1999–2011 Abbreviations: VAS, vitamin A supplementation; DPT3, 3^rd^ dose of the diphtheria, pertussis, and tetanus vaccine.

Across countries, institutional support, leadership, and ownership have been crucial to the success of MCHWs.^18^-^19^ In Sierra Leone, the MoHS and partners show their support and leadership by mobilizing resources to provide a platform for the campaign. In addition, launching the MCHWs at national, chiefdom, and zonal levels provides the opportunity to solicit the participation of politicians and opinion, traditional, and religious leaders.

### Barriers to VAS Receipt

Only a small number of children in Sierra Leone do not receive VAS services during MCHWs. The 2011 PEC survey identified lack of caregiver awareness of the MCHW event and lack of household visits by CHWs as barriers to VAS receipt. These barriers are similar to those found in the 2004 PEC survey, suggesting that additional targeted strategies are needed to deliver the MCHW package of services to these last hard-to-reach populations.

In addition, caregivers' knowledge of the impact of VAS on mortality needs to be strengthened. Increasing caregivers' awareness of VAS benefits and ensuring that key information on the MCHW events is disseminated may increase demand for these services. From the PEC survey, suggested effective forms of communication include radio broadcasting and traditional methods, including message dissemination through HWs and CHWs.

### Experiences With Mobile Phone Data Entry

We had a very positive experience using EpiSurveyor for the PEC survey, as it allowed the investigators to view raw data and summary statistics in real time, address questionable data while enumerators were still in the field, and quickly prepare the final report since the time needed for data entry was greatly reduced. Over 99% of the data entered with EpiSurveyor matched data from the paper surveys.

### Limitations

EA selection for the PEC survey was based on the 2004 national census—the most recent comprehensive population count for Sierra Leone available at the time. But the census data are widely acknowledged to be unrepresentative of the post-conflict situation, which includes rapid urbanization, unmonitored settlement of internally displaced people, and recent labor migrations due mostly to the mining sector.^20^ As a result, random selection of EAs was likely based on imprecise data, potentially leading to the selection of clusters with more populations that have migrated and the selection of fewer clusters in recently urbanized areas. Since 2012, it has become accepted at the national level to use a census conducted for mass drug administration to adjust population projections for VAS campaigns.

## CONCLUSIONS

High VAS coverage has been achieved in Sierra Leone through twice-yearly delivery of VAS through MCHW events. The MCHW events have driven equitable access to VAS and other essential child survival services in Sierra Leone. Results of the PEC survey indicate that outdated population data have led to higher VAS coverage reported through tally sheets and suggest that population data be reviewed to improve supply procurement estimates and increase the quality of coverage estimates.

The 2011 PEC survey confirms the major improvements in VAS coverage recorded between 2004 and 2005. Coverage in all districts was high, which demonstrates consistent quality of MCHW implementation nationally. Efforts to increase coverage among hard-to-reach populations should focus on increasing awareness of MCHW events and the impact of VAS on mortality.

High coverage of VAS and other essential child survival interventions through MCHW events has most likely contributed to the reduction in child mortality observed in Sierra Leone since 2005. Continuing the trajectory of high and equitable coverage of VAS through the MCHW events may enable Sierra Leone to achieve Millennium Development Goal 4: reducing child mortality by two-thirds to 90/1,000 by 2015.^21^-^22^
